# Genetic Alterations, DNA Methylation, Alloantibodies and Phenotypic Heterogeneity in Type III von Willebrand Disease

**DOI:** 10.3390/genes13060971

**Published:** 2022-05-28

**Authors:** Muhammad Asif Naveed, Aiysha Abid, Nadir Ali, Yaqoob Hassan, Ali Amar, Aymen Javed, Khansa Qamar, Ghulam Mustafa, Ali Raza, Umera Saleem, Shabbir Hussain, Madiha Shakoor, Shagufta Khaliq, Shahida Mohsin

**Affiliations:** 1 Department of Haematology, University of Health Sciences, Khayaban Jamia Punjab, Lahore 52600, Pakistan; khansaqamar@gmail.com (K.Q.); gmustafa798@gmail.com (G.M.); shahida.mohsin@yahoo.co.uk (S.M.); 2 Centre for Human Genetics and Molecular Medicine, Sindh Institute of Urology Transplantation, Karachi 42000, Pakistan; aiyshaabid@gmail.com (A.A.); raza.a005@gmail.com (A.R.); 3 Kulsoom International Hospital, 2020 Jinnah Ave, G 6/2 Blue Area, Islamabad 53000, Pakistan; dr.nadir.ali@gmail.com; 4 Chughtai’s Lahore Lab, 42300 Jail Road, Lahore 52600, Pakistan; hafizmyh.74d@gmail.com; 5 Human Genetics and Molecular Biology, University of Health Sciences, Khayaban Jamia Punjab, Lahore 52600, Pakistan; ali.amar@uhs.edu.pk (A.A.); madihashakoor1@gmail.com (M.S.); khaliq.shagufta@gmail.com (S.K.); 6 Department of Obstetrics and Gynaecology Services Hospital Jail Road, Lahore 42500, Pakistan; aymenasif86@gmail.com; 7 Department of Pathology, Nishter Medical University, Nishter Road, Multan 32003, Pakistan; umera_saleem@yahoo.com; 8 Department of Biochemistry, University of Health Sciences, Khayaban Jamia Punjab, Lahore 52600, Pakistan; ashas74040@gmail.com

**Keywords:** von Willebrand factor, von Willebrand disease, mutations, methylation, phenotypic heterogeneity

## Abstract

Type III von Willebrand disease is present in the Punjab province of Pakistan along with other inherited bleeding disorders like hemophilia. Cousin marriages are very common in Pakistan so genetic studies help to establish protocols for screening, especially at the antenatal level. Factors behind the phenotypic variation of the severity of bleeding in type III vWD are largely unknown. The study was conducted to determine Mutations/genetic alterations in type III von Willebrand disease and also to determine the association of different mutations, methylation status, ITGA2B/B3 mutations and alloimmunization with the severity of type III vWD. After informed consent and detailed history of the patients, routine tests and DNA extraction from blood, mutational analysis was performed by Next Generation Sequencing on Ion Torrent PGM. DNA methylation status was also checked with the help of PCR. In our cohort, 55 cases were detected with pathogenic mutations. A total of 27 different mutations were identified in 55 solved cases; 16 (59.2%) were novel. The mean bleeding score in truncating mutations and essential splice site mutations was relatively higher than weak and strong missense mutations. The mean bleeding score showed insignificant variation for different DNA methylation statuses of the *VWF* gene at the cg23551979 CpG site. Mutations in exons 7,10, 25, 28, 31, 43, and intron 41 splice site account for 75% of the mutations.

## 1. Introduction

von Willebrand disease (vWD) is an inherited bleeding disorder that is caused by a defect of von Willebrand factor (*VWF*), a glycoprotein important for platelet adhesion to the subendothelium after vascular injury [1]. Easy bruising, epistaxis and prolonged bleeding after surgery are the most common clinical manifestations. vWD is divided into three types. Type I and II are relatively less severe and are inherited in an autosomal dominant manner [2].

Type III vWD is a severe bleeding disorder inherited in a recessive manner in which there are negligible levels i.e., <5 IU/dL of plasma *VWF* leading to reduced factor (F) VIII levels [3]. Type III vWD is the rarest among vWD in International data [4,5]. Surprisingly, Pakistani data about the prevalence of different types of von Willebrand Disease shows that Type III is the most common type. According to Borhany et al., the prevalence of Type III vWD is 51.6% and that of type I and II remains 19.1% and 29.4%, respectively and according to Khan et al. type III was commonest with 94.1% followed by Type II and Type I with 3.92% and 1.96% respectively [6]. The gene of *VWF* is located on chromosome 12p having 52 exons encoding a protein of 2813 amino acids [7]. Worldwide studies have revealed that mutations are spread throughout the entire span of genes and few studies have reported that mutations in certain sites are more prevalent in a particular population [8,9,10,11]. In addition to the inherited form, several other forms of the disease have been reported, including autoantibodies against *VWF* and conditions that may cause rapid degradation and clearance of *VWF* and adsorption of *VWF* on malignant cells. This kind of disease is called acquired von Willebrand syndrome (AVWS) [12]. Studies have also reported that alloimmunization in patients of type III vWD may also lead to clearance of *VWF*. In patients with alloantibodies, subsequent treatment with *VWF* concentrates is not only ineffective, but may cause post-infusion severe allergic reactions like abdominal pain, lumbar pain, hypotension, and anaphylactic shock, because of the formation of immune complexes that activate the complement system [13].

Grey areas exist between symptomatic type I vWD and less severe type III vWD which needs exploration at the genetic level for clinical decision making. There is considerable variation in the mutation spectrum from one country to another and one region to other. The only available study reported from Sindh Province, Pakistan, showed 19 novel mutations [8]. Due to variation in data, there is a need for a local study to identify genetic alterations, which in turn will help in devising customized gene panel and antenatal diagnosis. There is also considerable phenotypic variation in bleeding severity of the type III vWD patients ranging from less severe to markedly severe bleeding. The underlying factors of this phenotypic heterogeneity have not been explored. All these things urge the need to explore vWD from different angles including exploration at the genetic level, methylation status, ITGA2B/B3 and alloimmunization.

Considering all the above facts, the objectives of this study were to determine the mutations/genetic alterations of type III von Willebrand disease and also to determine the association of different mutations, DNA methylation status, ITGA2B/B3 mutations and alloimmunization with the severity of type III vWD.

## 2. Patients and Methods

### 2.1. Operational Definitions

von Willebrand Disease Type I: vWF:Ag and Ricof level less than 30 IU/dL.

von Willebrand Disease Type II: vWF:Ag > 50 IU/dL and Ricof level less than 30 IU/dL with Ricof/vWF:Ag ratio of <5 [14,15,16].

von Willebrand Disease Type III: vWF:Ag and Ricof less than 5 IU/dL.

Thrombocytopenia: Platelet count less than 150 × 10^9^/L.

### 2.2. Setting and Study Design

The study was conducted from July 2019 to June 2021 in accordance with the Declaration of Helsinki, and approved by the Ethics Committee of the University of Health Sciences, Lahore 224/20-8-2018. The study was conducted at the Department of Human Genetics and Molecular Medicine Sindh Institute of Urology and Transplantation Karachi, Department of Human Genetics and Molecular Biology University of Health Sciences Lahore and Department of Haematology University of Health Sciences, Lahore.

It was a cross-sectional study using convenient sampling technique and WHO calculator 2.0.2.21 was used for sampling technique. All patients diagnosed with type III von Willebrand disease were enrolled in the study. Patients with personal/family history of bleeding disorders other than the von Willebrand disease and patients with comorbidities were excluded.

### 2.3. Sample Collection and Storage

Patients meeting the inclusion criteria were enrolled in the study. The data of diagnosed cases of von Willebrand disease were taken from the Hemophilia Society of Lahore. An extensive search was done for the enrolment of patients in the study. The research team visited many cities of Punjab (i.e., Sargodha, Faisalabad, Bahawal Nagar, Multan, Rawalpindi, Gujrat, Sialkot, Dera Ghazi Khan, Khan Pur and Sahiwal) to collect the clinical data and samples of the patients. The workflow used in the study has been explained in Supplementary Figure S1.

For bias reduction, only unrelated patients were enrolled and a questionnaire was filled out by an independent hematologist (not part of the research team).

### 2.4. Patients

Informed consent was obtained from all subjects involved in the study and 71 unrelated patients of all ages having type III vWD were included in the study. Written informed consent was also obtained from the patient to publish this paper after the research. These patients were also confirmed in Haematology Lab for diagnosis on the basis of Platelet count, Bleeding Time (BT), PT (Prothrombin Time), Activated partial thromboplastin time (APTT), FVIII levels and *VWF*: Ricof levels (Ristocetin induced co-factor activity). The procedure of these tests is explained in the supplementary data file having the title P1-6. After informed consent, detailed history of the patients was taken and noted on the prescribed proforma, i.e., Condensed MCMDM-1 bleeding questionnaire [17] also attached as supplementary file.

### 2.5. Genetic Analysis

DNA extraction: Genomic DNA was extracted from EDTA-anticoagulated peripheral blood samples using the standard Phenol-Chloroform extraction method with some modifications [18].

Gene panel sequencing: We used a custom-designed gene panel targeting all coding exons and adjacent splice sites of 03 genes (a total of 97 exons) that are known to cause the most common inherited bleeding disorders including vWD (*VWF* gene) and Glanzmann Thrombasthenia (ITGA2B and ITGB3 genes).

The NGS libraries were prepared from 20–25 ng of DNA per sample using the Ion Ampliseq library kit v2.0 (Life Technologies, Carlsbad, CA, USA) according to the manufacturer’s protocol. Briefly, after PCR amplification of target regions, NGS libraries were adapter-ligated using the Ion Xpress barcode adapter kit (Life Technologies, Carlsbad, CA, USA) for multiplexing in a single NGS run and were subsequently purified using the Agencourt AMPure XP reagent kit (Beckman Coulter, Brea, CA, USA). Next, the quantified libraries (by using a qPCR quantitation kit or Qubit dsDNA HS assay kit) were subjected to template preparation and clonal amplification by emulsion PCR using Ion PGM Template OT2 200 kit (Life Technologies, Carlsbad, CA USA) according to the manufacturer’s protocol [19,20]. The samples were then sequenced on an Ion Torrent PGM system (Life Technologies, CA, USA) using the Ion PGM sequencing kit v2 and an Ion 314 chip kit v2 (Life Technologies, Carlsbad, CA, USA) with 500-flows PGM run [19,20].

### 2.6. Mutational Data Analysis

The raw gene panel sequencing data were processed for trimming of barcode adapters, base calling, filtering, signal analysis including removal of poor signal-profile reads and other QC analyses using the Torrent Suite v5.6.0 software package (Life Technologies, Carlsbad, CA, USA). Cleaned reads/sequences were mapped to the human reference genome assembly GRCh37 (hg19). Subsequently, base recalibration, indel realignment and variant calling were done using the Torrent Variant Caller plugin (v5.6.0.5), whereas the number of mapped reads, the percentage of on-target reads, and the mean depth of reads were determined using the Coverage Analysis Plugin (v5.0.4.0).

Annotation and filtration of the variants called were performed using Ion Reporter Software (v5.6; Life Technologies, Carlsbad, CA USA) and Alamut Visual (v 1.4.4 Interactive Biosoftware, Boston, MA, USA) following a previously published protocol [21]. Briefly, only small INDELs and non-synonymous (exonic) and splice site variants were retained in the first step, thus excluding any synonymous and intronic variants outside the splice site regions. Then allele frequencies were used to filter out variants that were too common in the population (>1 in 10,000) using dbSNP150 database in the second step, thus ensuring the selection of only rare variants. In the third step, prioritization of remaining variants was performed considering their potential impact on the structure/function of the encoded protein, especially for the novel mutations. Truncating mutations (such as stop and frameshift loss of function mutations) and obligatory splice site mutations were considered likely pathogenic. Prioritization of missense mutations was based on the use of different in silico pathogenicity prediction tools including PolyPhen-2 [22], SIFT [23] and Mutation Taster [24] and also by constructing multiple sequence alignments (MSA) of orthologs across phylogeny to determine evolutionary conservation. In addition, variants that were frequented in homozygous states in healthy control datasets such as Genome Aggregation Database (gnomAD) were also excluded. By default, all variants implicated as causative mutations in individuals with vWD in earlier studies (as determined by a review of existing literature) were taken as potentially pathogenic. The gene symbols and sequence variants were defined according to the recommendations of the Human Genome Organization (HUGO) human gene nomenclature committee, available at https://www.genenames.org/ (accessed on 3 March 2022), and Human Genome Variation Society (HGVS) sequence variant nomenclature available at https://varnomen.hgvs.org/ (accessed on 1 March 2022), respectively.

### 2.7. Sanger Sequencing

After putative variants were identified, Sanger sequencing was used to validate the variants in original DNA samples of patients. Primers used for this purpose were either self-designed or as reported by Mohl A et al. [25]. These primer sequences are given in Supplementary Table S1. Sequence chromatograms from patient samples were visualized using Chromas v2.6.6 (http://technelysium.com.au/wp/chromas/, accessed on 3 March 2022) and compared with reference gene sequence using blastn suite (https://blast.ncbi.nlm.nih.gov/, accessed on 3 March 2022) to map and confirm the presence and zygosity state of mutations detected in gene panel sequencing.

### 2.8. In Silico Structural Analysis

In order to predict their putative structural impact, protein modeling and visualization of the novel or previously uncharacterized *VWF* missense and truncating mutations were performed. In the absence of an experimentally determined biophysical protein structure of *VWF* protein domains D1-D2, SWISS-MODEL server (https://swissmodel.expasy.org/, accessed on 3 March 2022) was used for homology protein modelling of wild-type *VWF* protein domains D1–D2. While pdb files for individual crystal structures of other *VWF* domains including D′-D3 (PDB ID: 6N29), A1 (PDB ID: 1AUQ), A2 (PDB ID: 3GXB), and A3 (PDB ID: 1AO3) domains were downloaded from RCSB PDB (https://www.rcsb.org/, accessed on 3 March 2022). Target mutations were introduced using the BIOVIA Discovery Studio Visualizer (Dassault Systèmes, Vélizy-Villacoublay, Île-de-France, France) and wild type as well as mutant structures were visualized and images rendered with PyMOL Molecular Graphics System, Schrödinger, LLC (http://www.pymol.org, accessed on 3 March 2022).

### 2.9. DNA Methylation Analysis

Significant *VWF* CpG sites that were reported to influence the DNA methylation status dependent expression of *VWF* gene, known as expression quantitative trait methylation (eQTMs), were identified from BIOS QTL browser (https://genenetwork.nl/biosqtlbrowser/, accessed on 7 March 2022). The two most significant eQTMs (cg23551979 and cg04053108) located in/near a *VWF* CpG island region were selected for DNA methylation status analysis. Two MSP primer sets were designed to amplify the target CpG containing region of *VWF* gene using the MethPrimer tool (available at https://www.urogene.org/cgi-bin/methprimer, accessed on 9 March 2022), allowing for methylation status dependent specific amplification of methylated and un-methylated alleles by methylated and unmethylated primers, respectively. The primer sequences are given in Supplementary Table S2. An overview of the target *VWF* CpG sites covered and MSP primers designed is presented in Supplementary Figure S2.

Bisulfite conversion of 200–500 ng of genomic DNA for each sample was done using EpiJET Bisulfite Conversion Kit (ThermoFischer Scientific, Waltham, MA, USA) following the manufacturer protocol. The methylation-specific PCR (MSP) reaction was performed using DreamTaq Hot Start PCR Master Mix (Thermo Scientific, Waltham, MA, USA) following standard PCR reaction conditions and thermal cycling with each batch including human methylated and non-methylated DNA standards (Zymo Research, Irvine, CA, USA) as well as non-converted genomic DNA and No-Template-Control for quality control purposes. The MSP products were visualized on 2% agarose gels under UV-light using Gel Doc system (BIO-RAD, Hercules, CA, USA) and based on the results, the methylation status of each sample was determined as methylated, partially methylated or unmethylated.

### 2.10. Statistical Analysis

Statistical analyses were performed using either IBM SPSS Statistics, version 24 or GraphPad Prism version 8. After assessing the distribution, data for quantitative variables were presented as either mean ± standard deviation (normally distributed data such as bleeding score) or median and range (min-max) (non-normally distributed data such as *VWF* antigen levels) and compared using Mann-Whitney U or Kruskal-Wallis and Student’s *t*-test or ANOVA or linear trend test (as appropriate), respectively. While qualitative variables (frequency data such as DNA methylation status) were given as a number (percentage) and their comparison and evaluation of group relationships involved Fisher’s exact test or the chi-square (χ^2^) test as applicable. A *p*-value of ≤0.05 was considered statistically significant in all comparisons made unless stated otherwise.

## 3. Results

A total of 71 patients with type III von Willebrand Disease were enrolled. Out of these 71 patients, the male to female ratio was 1.1:0.9 (37/34) and the median age was 19 years (range 3–66 years and IQR of 15 Years).

Bleeding score was measured by the Condensed MCMDM1 tool and found to be 15.87 ± 4.414. Bleeding scores in males and females were 14.86 ± 3.735 and 16.98 ± 4.871, respectively. A total of 47 (66%) patients had a family history of prolonged bleeding, while 24 (34%) patients had no such family history. Mutation detection frequency was compared between genders, presence/absence of family history of bleeding, presence/absence of alloantibodies and >15 min/<15 min of bleeding time as explained in Supplementary Table S3A–D.

A total of 5 (7%) patients were detected with alloantibodies against von Willebrand Factor, while no alloantibody against von Willebrand Factor was detected in 66 (93%) patients. Three of these five patients were females, while 2 were males.

A total of 55 (77.5%) patients had homozygous, compound heterozygous, or isolated heterozygous mutation(s) detected, while 16 (22.5%) patients remained unsolved, showing no mutation. The mutation was detected in 27 (72.9%) out of 37 males and 28 (82.34%) out of 34 females. Out of the total 55 solved cases, 27 (49.1%) were males and 28 (50.9%) were females; this difference was not statistically significant by the Chi-Square test with *p*-value of 0.51. Out of the total 55 solved cases, 35 (63.6%) patients had a family history of prolonged bleeding, and 20 (36.4%) patients had no such family history (statistically not significant).

**Figure 1:**
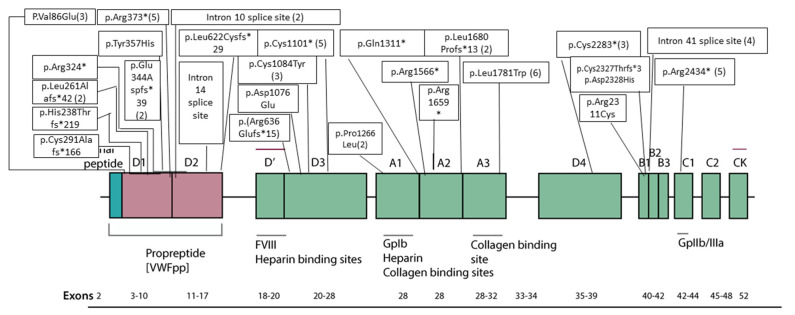
Mutations in different regions of *VWF* gene with respect to exon, domains of protein and bindings sites. The lowest line shows the exon arrangement. Above than the exon arrangement functional binding sites/protein. Domains of the *VWF* gene are represented in color boxes. Amino acid changes, truncations and splice site mutations are expressed in plain boxes. Number of patients having particular mutation is given in brackets against each amino acid change, termination or splice site mutations.

Out of 55 solved cases of type III vWD, 49 patients had homozygote/compound heterozygote mutations while 6 patients had isolated heterozygote mutations. Mutations in different regions of *VWF* gene with respect to exon, domains of protein and bindings sites are explained in Figure 1.

**Figure 2:**
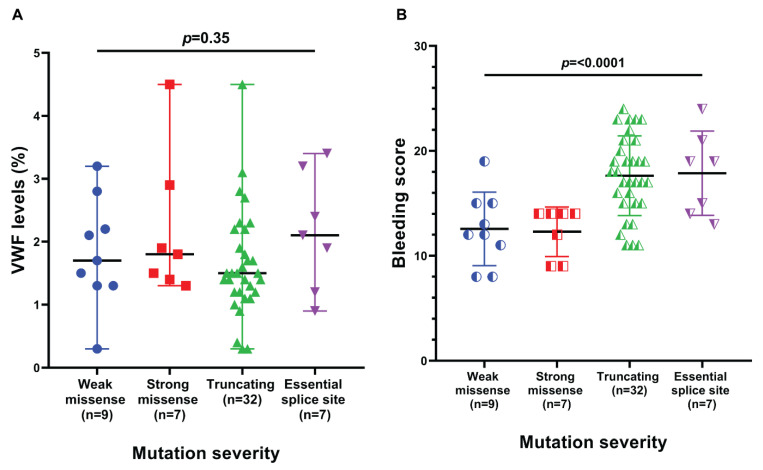
Genotype–phenotype correlations between *VWF* mutation severity and (**A**) *VWF* antigen levels, and (**B**) bleeding score in Pakistani vWD patients.

**Figure 3:**
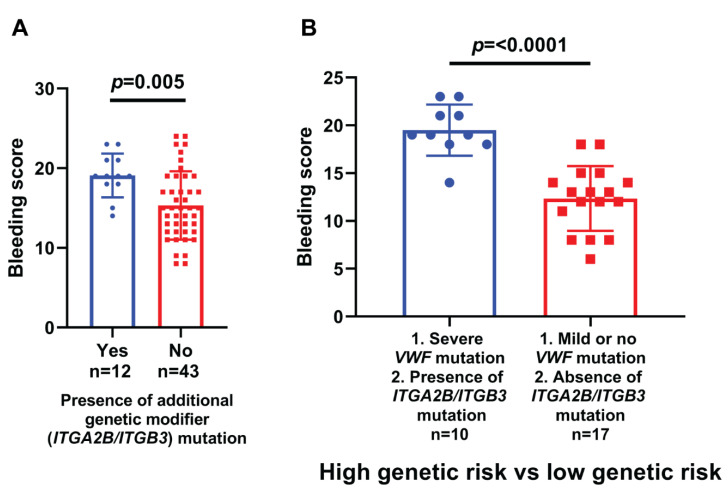
Bleeding score in Pakistani vWD patients stratified by (**A**) presence of additional genetic modifier (ITGA2B/ITGB3) mutation in addition to the causative *VWF* mutation, and (**B**) high genetic risk vs. low genetic risk.

In our cohort, 27 different mutations were identified in 55 solved cases; 16 (59.2%) out of these 27 mutations were novel. These 16 mutations were present in 26 patients as shown in Table 1. Genotype-phenotype correlation is explained in Figure 2 and Figure 3. A representative Sanger confirmation electropherogram is attached in supplementary Figure S3 and  Figure S4. Demographic, clinical and laboratory features of vWD patients across *VWF* mutation detection status are explained in supplementary Figure S5.

### 3.1. D protein Structural Analysis

**Figure 4:**
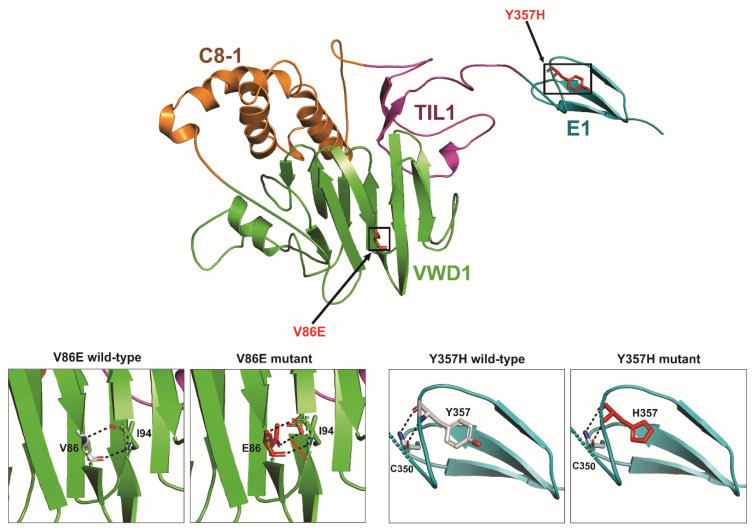
The 3D protein modelling of *VWF* p.Val86Glu and p.Tyr357His pathogenic variants. The predicted crystal structure of human *VWF* protein (D1 domain) is presented on the top. The modular domain organization features highlighted as von Willebrand D domain (vWD in green), 8 cysteines (C8 in orange), trypsin inhibitor-like (TIL in magenta), and E (in analogy to the A, C, and D repeats in *VWF* in cyan). The black open squares locate the p.V86E and p.Y357H mutations in the vWD1 and E1 domains, respectively. The inserts on the lower left depict the aberrant polar contacts induced by p.V86E mutation where amino acid residues involved in polar contacts are shown as stick models with carbon atoms for V86, E86 and I94 in grey, red and green, respectively. Likewise, the inserts on the lower right indicate conformational changes resulting from p.Y357H mutation.

**Figure 5:**
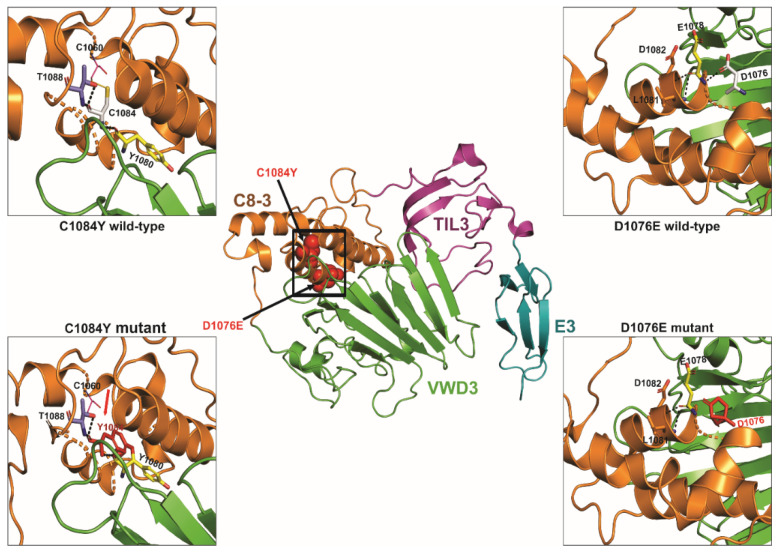
Ribbon diagrams highlighting p.Cys1084Tyr and p.Asp1076Glu mutations in the D3 domain of *VWF* protein. The experimentally determined 3D protein structure of human *VWF* D3 domain is shown in the middle with modular domains highlighted as vWD3 (green), C8-3 (orange), TIL3 (magenta), and E3 (cyan). The black open square locates the p.C1084Y and p.D1076E mutations, both in the vWD3 domain. The inserts on the left depict the aberrant disulfide bonding as a result of p.C1084Y mutation, whereas the inserts on the right show changes in hydrogen bonding consequential of p.Y357H mutation. The amino acid residues involved in disulfide bonding and polar contacts are shown as stick models. Models were obtained with PyMol Molecular Graphics System and 6N29 file.

**Figure 6:**
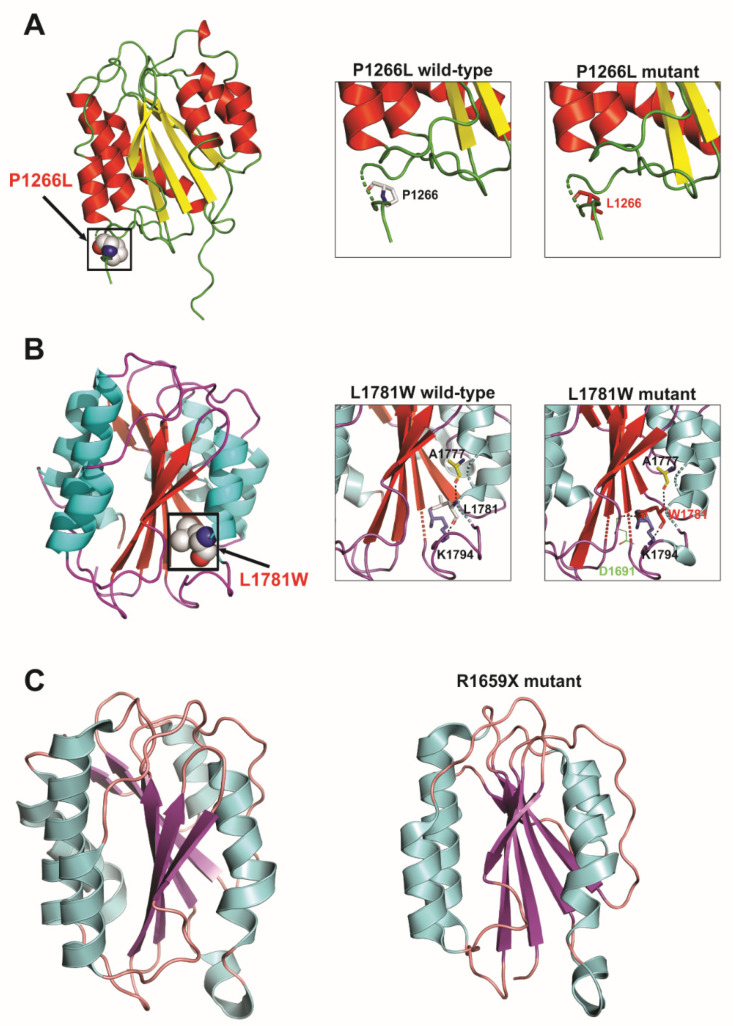
Cartoon representations of selected missense and truncating mutations in the *VWF* A1, A2 and A3 domains. (**A**) Experimentally determined 3D protein structure of human *VWF* A1 domain (left) and localization as well as steric encumbrance caused by the p.Pro1266Leu amino acid substitution (inserts on the right). (**B**) Ribbon diagram of the entire *VWF* A3 domain (left) and in small windows on the right are enlarged views of p.Leu1781Trp mutation illustrated as graphical sticks with changes in H-bonding (black dotted lines). A potential H-bond (with D1691) introduced by the presence of W1781 is highlighted. (**C**) Loss of secondary structure elements and changes in the overall 3D protein structure for truncating p.Arg1659Ter mutant are observed upon comparison of the wild-type (left) and mutant (right) *VWF* A2 domain protein structures. Models were obtained with PyMol Molecular Graphics System and 1AUQ (A1 domain), 1AO3 (A3 domain) and 3GXB (A2 domain) pdb files.

We also performed 3D protein modelling analyses to assess the potentially deleterious effects of a few selected novel or previously uncharacterized *VWF* missense and truncating mutations identified in this study. These analyses revealed that *VWF* p.V86E (D1 domain), p.D1076E (D3 domain) and p.L1781W (A3 domain) all cause aberrant hydrogen bonding resulting in steric encumbrance and conformational changes while p.C1084Y (D3 domain) disrupts a conserved disulfide bond (C1084–C1060) and results in the introduction of a free cysteine residue, adding further to mutation associated conformational changes in overall domain structure. In addition, the p.R1659X mutation resulted in premature truncated protein of 1659 amino acid residues instead of 2813 residues and direct comparison of this truncated mutant and wild-type *VWF* protein indicated loss of significant secondary structures and also showed conformational changes, which altered the overall tertiary as well as quaternary structure of the affected A2 domain and *VWF* protein, respectively. However, since p.Y357H and p.P1266L mutations localize to loop structures in *VWF* D1 and A1 domains, respectively, their effect on overall 3D structural conformation is less pronounced and may require cell biology based functional assays in supporting their pathogenic nature. The results of these protein modeling analyses are illustrated in Figure 4, Figure 5 and Figure 6.

### 3.2. DNA Methylation Status

**Figure 7:**
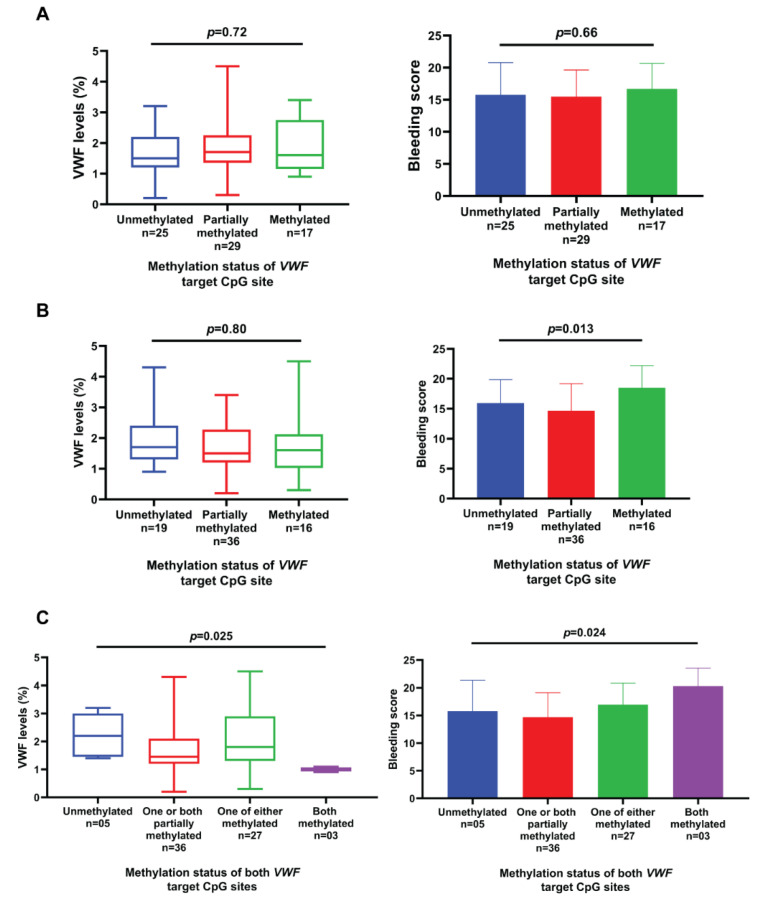
(**A**) *VWF* levels and mean bleeding score according to the DNA methylation status of *VWF* gene cg23551979 CpG site in Type III vWD patients. (**B**) *VWF* levels and mean bleeding score according to the DNA methylation status of *VWF* gene cg04053108 CpG site in Type III vWD patients. (**C**) *VWF* levels and mean bleeding score according to the combined DNA methylation status of *VWF* gene cg23551979 and cg04053108 CpG sites in type III vWD patients. The middle line of the box plot shows the median, the upper line of the box represents the 75th percentile, the lower line of the box represents the 25th percentile and the whisker below and above Q1 and Q4, respectively. The upper limit and whisker of blue, red, purple and green boxes represent the mean bleeding score and SD in patients having unmethylated, partially methylated and methylated CpG sites, respectively.

DNA methylation status of the *VWF* gene at cg04053108 and cg23551979 CpG site was checked and it was found that 19 (26.7%) and 27 (38%) were unmethylated, 36 (50.7%) and 29 (41%) patients had partial methylation, whereas 16 (22.5%) and 15 (21%) patients were methylated at these sites respectively. The mean bleeding score at the cg04053108 site was significantly different (*p*-value 0.013) when compared with reference to methylation status. When we compared *VWF* levels and mean bleeding time to DNA methylation status (methylated, unmethylated and partially methylated) of both CpG sites (cg04053108 and cg23551979), the difference was significant with a *p*-value of 0.025 and 0.024 respectively as explained in Figure 7.

## 4. Discussion

Type III vWD is considered the most severe form of von Willebrand Disease. Studies conducted in different regions of the world have shown different mutations [8,9,10,11]. Scarce data is available regarding the Pakistani Population. Type III is largely known as autosomal recessive disease, but there is a Canadian study that demonstrated a co-dominant model as well [26]. We analyzed a cohort of 71 un-related type III vWD patients. Out of these 71 patients, the male to female ratio was 1.1:0.9 (37/34) and the median age was 19 years (range 3–66 years and IQR of 15 Years). The study conducted by Ahmad et al. demonstrated that 26 (54%) were males, and 22 (46%) were females. The mean age was 11.5 ± 4.1, with the upper range only 22, while in our cohort, it was 66 [27].

In our study, a total of 5 (7%) patients were detected to have alloantibodies against von Willebrand Factor. Different studies have reported different incidences of alloantibodies ranging from 7.5% to 9.5%, whereas a study from Pakistan did not report any alloantibody formation [26,27]. A study from India reported 2 out of 77 patients had alloantibodies [9]. There is a considerable difference in prevalence in different studies, which raises the need to investigate underlying genetic or environmental factors. This study explored genetic factors underlying alloantibodies, but no association was found.

Different studies have identified mutations in 90–100% of patients while in our study, 55 (77.5%) patients were found to have mutation(s), while 16 (22.5%) patients remained unsolved and showed no mutation ([9,10,26,27]). This may be due to the presence of large deletions/insertions and complex rearrangements in our populations, for which further investigations are needed as NGS may miss such genetic changes; researchers are using MLPA to address these issues [28]. Another fact is that different studies have reported many cases that have apparent phenotypes of type III vWD, but no *VWF* mutation was identified after evaluation of entire protein-coding and flanking intronic regions [29]. This is the reason ISTH recommends failure to identify a causative mutation in the *VWF* gene does not rule out von Willebrand Disease [30].

A total of 9 (12.6%) patients in our cohort had weak missense mutations, 7 had strong missense mutations (9.8%), 32 (45.1%) had truncating mutations and essential splice site mutations were found in 7 (9.8%) patients. The results of Ahmad et al. were similar to our study as they comprised 27 missense mutations (~30%), 23 nonsense mutations (~25%), 22 gene conversions (~23%), 10 splice site alterations (~11%), 5 small insertions (~5%), 4 small deletions (~4%) and 2 large deletions (~2%) [27]. Another study conducted by Kasatkar et al. also showed similar results [9]. In our study, no large deletion was discovered. Detection of large deletions needs MLPA or dedicated variant calling pipeline for NGS data; unsolved cases need investigation by these techniques for the possibility of large deletions.

In our cohort, 27 different mutations were identified in 55 solved cases; 16 (59.2%) out of these 27 mutations were novel. These 16 mutations were present in 26 patients. Quite a few studies have been conducted in the 21st century exploring genetic alterations in type III von Willebrand Disease. In 2003, Broniciani et al. found 45 novel mutations out of 50 total mutations while in 2014, Kataskar in India found 34 (57.6%) novel mutations in 59 total mutations, whereas, in 2013, Bowman et al. showed 20 (64.5%) novel mutations out of 31 [9,26,31].

The mean bleeding score in truncating mutations and essential splice site mutations was relatively higher than weak and strong missense mutations. This difference in mean bleeding score was significant. It means that patients with splice site mutations and truncating mutations have a more severe phenotype as compared to weak and strong missense mutations despite there not being any statistically significant difference in *VWF* antigen levels. Coagulation is a complex phenomenon in which vessel wall, platelets, coagulation factors, coagulation co-factors and von Willebrand Factor are involved. When *VWF* is formed, it passes through different stages like histone modification, storage and release of *VWF*. One of the above factors may have contributed to the increase in bleeding scores of the patients. One study reported that *VWF* antigen levels vary in members of a single-family, raising the possibility of other genetic modifiers [32]. We were unable to find any other study that compared the bleeding score with the type of mutation.

We also analyzed the mutations in ITGA2B and ITGB3 in these patients as possible modifiers of the phenotype of the disease. We found that 18 (25.3%) patients have mutations in at least one of the genes. These 18 patients have 10 types of mutations in ITGA2B and ITGB3 genes. The mean bleeding score in solved type III vWD cases having ITGA2B/ITGB3 mutations was higher as compared to solved type III vWD cases not having mutations in the ITGA2B/ITGB3 gene. This means that genetic variants/mutations in other hemostatic factors affect the phenotype of the type III vWD. No other study has compared the phenotype of type III vWD with respect to ITGA2B/ITGB3 mutations.

Current management guidelines advocate prophylaxis based on the history of bleeding only, which may predispose a patient to life-threatening bleeding, especially at an early age [33]. We proposed two risk groups; a high-risk group having severe (truncating or splice-site mutations with the presence of the mutation in ITGA2B/ITGB3 gene) and a low-risk group (having mild or no *VWF* mutations with the absence of mutation in ITGA2B/ITGB3 gene). The high-risk group had a more severe phenotype with a significantly high mean bleeding score.

We also checked the DNA methylation status of the *VWF* gene at cg04053108 CpG and cg23551979 CpG sites and compared methylated, partially methylated and unmethylated groups for the mean bleeding score and vWF:Ag. The aim was to see if methylation status affects the phenotype of the disease but the difference in mean bleeding score and vWF:Ag of the three groups was not statistically significant at cg23551979 CpG while methylation of cg04053108 CpG site was associated with an increase in bleeding time consistent with more severe phenotype.

We further analyzed a few selected novel or previously uncharacterized *VWF* missense and truncating mutations by 3D protein modelling. Among these, p.V86E and p.Y357H mutations localized to *VWF* prodomain D1 (involved in mediating intermolecular disulfide bridges and intracellular storage of *VWF* protein). These mutations (particularly p.V86E by affecting structured H-bonding) are predicted to influence the maturation, multimerization and secretion of *VWF* protein. Indeed, p.V86E demonstrated severely impaired secretion of mutant *VWF* protein, suggesting its intracellular retention in transient expression experiments using HEK293T cells [34]. Similarly, *VWF* protein multimers are formed in the Golgi due to the N-terminal disulfide bonding between cysteines in the D3 domains [35]. Therefore, the novel D3 domain missense mutation identified in this study (p.D1076E and particularly p.C1084Y, which causes aberrant disulfide bonding) is predicted to affect the multimerization process and also FVIII binding capacity, thus hindering the normal functioning of *VWF* mature protein, manifesting as vWD in patients harboring them [36]. Likewise, p.P1266L in the A1 domain, p.R1659X in the A2 domain and p.L1781W in the A3 domain are predicted to disrupt GpIb binding, *VWF* length regulation and binding of collagen type I and III, respectively [37]. Among these, the case of p.P1266L is particularly interesting. Although analysis of the resolved crystal structure of the *VWF* A1 domain localizes p.P1266L mutation to a loop region with minimal resultant conformational changes in domain structure, p.P1266L has previously been reported as a gain of function variant with enhanced *VWF* binding to platelets GPIb and resulting in type-2B vWD [34]. Altogether, the reported pathogenic mutants may cause their deleterious effects by influencing various steps in *VWF* biogenesis or physiology, manifesting ultimately as vWD.

Although mutations of *VWF* were found in almost the entire span of the gene, mutations in exons 7,10, 25, 28, 31, 43, and intron 41 splice site account for 75% of the mutations. Therefore, we hereby propose that as a first step, targeted sequencing for these exons may be carried out in difficult diagnostic and antenatal cases for appropriate and cost-effective clinical decision-making. A more comprehensive approach may be adopted if a pathogenic mutation is not found in these areas.

## 5. Conclusions

The type III vWD has considerable phenotypic variability. If clinical decision-making demands genetic testing for vWD, then a targeted approach for most commonly involved exons/area (exons 7,10, 25, 28, 31, 43, and intron 41) may be adopted considering the limitations of a resource-limited country. Methylation at cg04053108 CpG is associated with a more severe phenotype of the disease. The genetic workup of detection of causative mutation and assessment of other genes influencing the phenotype severity and environmental factors may help categorize high and low-risk groups earlier so that prophylactic treatment may be started early.

## Figures and Tables

**Table 1:** *VWF* gene mutations identified in Pakistani vWD patients with type III disease.

Family- Individual	Age (Years)	Sex	*VWF* Level IU/dL	Bleeding Score	Nucleotide Change	Protein Change	Type of Mutation	Exon/Intron and Zygosity	In Silico Severity Scores	Evolutionary Conservation	gnomAD (H/h/T)	rs ID	Reported/ Novel
vWD01	30	F	1.5	19	g.6180502G>A c.1117C>T	p.Arg373*	Nonsense	Ex.10/Hom	NA	NA	0/6/251032 (All) 0/4/30550(SA)	rs62643625	Reported
vWD02	29	F	1.1	23	g.6180502G>A c.1117C>T	p.Arg373*	Nonsense	Ex.10/Hom	NA	NA	0/6/251032 (All) 0/4/30550(SA)	rs62643625	Reported
vWD03	9	F	1.2	21	g.6091158C>T c.7082-1G>A	NA	Splice site	In. 41/Hom	Splice score—100%	NA	NR	NA	Novel (This study)
vWD04	17	F	0.9	24	g.6091158C>T c.7082-1G>A	NA	Splice site	In. 41/Hom	Splice score—100%	NA	NR	NA	Novel (This study)
vWD05	29	F	1.5	15	g.6166105delG c.1864delC	p.Leu622Cysfs*29	Frameshift	Ex. 15/Hom	NA	NA	NR	NA	Novel (This study)
vWD6	14	F	2.2	19	g.6094781G>T c.6849C>A	p.Cys2283*	Nonsense	Ex.39/Hom	NA	NA	NR	NA	Novel (This study)
vWD7	7	M	1.7	16	g.6094781G>T c.6849C>A	p.Cys2283*	Nonsense	Ex.39/Hom	NA	NA	NR	NA	Novel (This study)
vWD8	7	F	1.4	12	g.6127545delA c.5039delT	p.Leu1680Profs*13	Frameshift	Ex. 28/Hom	NA	NA	NR	NA	Novel (This study)
vWD9	7	M	1.5	23	g.6132873G>T c.3303C>A	p.Cys1101*	Nonsense	Ex.25/Hom	NA	NA	NR	NA	Bowman (2013) J Thromb Haemost 11, 512
vWD10	21	F	1.4	21	g.6128653G>A c.3931C>T	p.Gln1311*	Nonsense	Ex.28/Hom	NA	NA	0/13/250944 (All) 0/12/30604 (SA)	rs267607337	Casana (2000) Br J Haematol 111, 552 and others
vWD11	18	M	1.9	15	g.6091158C>T c.7082-1G>A	NA	Splice site	In. 41/Hom	Splice score—100%	NA	NR	NA	Novel (This study)
vWD14	26	M	3.2	19	g.6180462C>T c.1156+1G>A	NA	Splice site	In. 10/Het	Splice score—100%	NA	NR	NA	Novel (This study)
vWD15	24	M	2.1	13	g.6180462C>T c.1156+1G>A	NA	Splice site	In. 10/Het	Splice score—100%	NA	NR	NA	Novel (This study)
vWD18	8	M	0.3	18	g.6132873G>T c.3303C>A	p.Cys1101*	Nonsense	Ex.25/Hom	NA	NA	NR	NA	Bowman (2013) J Thromb Haemost 11, 512
vWD19	35	M	1.7	15	g.6127888G>A c.4696C>T	p.Arg1566*	Nonsense	Ex.28/Hom	NA	NA	0/2/125338 (All)	rs61750112	Yes (Indian + Chinese studies)
vWD20	11	F	1.9	9	g.6220098A>T c.257T>A	p.Val86Glu	Missense	Ex. 4/Hom	PPh2 = D SIFT = DL MT = DC	*Gallus gallus* *(G. gallus)*	NR	NA	Ahmed, 2019
vWD21	7	M	2.8	8	g.6125368A>C c.5342T>G	p.Leu1781Trp	Missense	Ex. 31/Hom	PPh2 = PD SIFT = DL MT = P	*Homo sapiens* *(H. sapiens)*	NR	NA	Ahmed, 2019
vWD22	26	F	0.3	11	g.6125368A>C c.5342T>G	p.Leu1781Trp	Missense	Ex. 31/Hom	PPh2 = PD SIFT = DL MT = P	*H. sapiens*	NR	NA	Ahmed, 2019
vWD23	17	M	3.2	12	g.6125368A>C c.5342T>G	p.Leu1781Trp	Missense	Ex. 31/Hom	PPh2 = PD SIFT = DL MT = P	*H. sapiens*	NR	NA	Ahmed, 2019
vWD25	20	M	1.6	17	g.6132873G>T c.3303C>A	p.Cys1101*	Nonsense	Ex.25/Hom	NA	NA	NR	NA	Bowman (2013) J Thromb Haemost 11, 512
vWD26	19	F	1	19	g.6132873G>T c.3303C>A	p.Cys1101*	Nonsense	Ex.25/Hom	NA	NA	NR	NA	Bowman (2013) J Thromb Haemost 11, 512
vWD27	17	M	1.2	11	g.6127609G>A c.4975C>T	p.Arg1659*	Nonsense	Ex.28/Het	NA	NA	0/16/281716 (All) 0/0/30562 (SA)	rs61750595	Zhang (1992) Hum Mol Genet 1, 61 and others
					g.6184595insC c.780InsG	p.Leu261Alafs*42	Frameshift	Ex. 7/Het	NA	NA	NR	rs760130928	Novel (This study)
vWD28	19	M	3.4	14	g.6091158C>T c.7082-1G>A	NA	Splice site	In. 41/Hom	Splice score—100%	NA	NR	NA	Novel (This study)
vWD29	20	F	1.9	13	g.6166066delC c.1905delC	p.Arg636Glufs*15	Frameshift	Ex. 15/Het	NA	NA	NR	NA	Novel (This study)
vWD30	13	M	3.1	15	g.6085414G>A c.7300C>T	p.Arg2434*	Nonsense	Ex. 43/Hom	NA	NA	0/2/31380 (All) NR (SA)	rs62643640	Baronciani (2003) Blood Cells Mol Dis 30, 264 and others
vWD32	30	F	1.5	8	g.6125368A>C c.5342T>G	p.Leu1781Trp	Missense	Ex. 31/Hom	PPh2 = PD SIFT = DL MT = P	*H. sapiens*	NR	NA	Ahmed, 2019
vWD33	29	M	4.5	14	g.6220098A>T c.257T>A	p.Val86Glu	Missense	Ex. 4/Hom	PPh2 = D SIFT = DL MT = DC	*G. gallus*	NR	NA	Ahmed, 2019
vWD35	35	M	4.5	24	g.6085414G>A c.7300C>T	p.Arg2434*	Nonsense	Ex. 43/Hom	NA	NA	0/2/31380 (All) NR (SA)	rs62643640	Baronciani (2003) Blood Cells Mol Dis 30, 264 and others
vWD36	34	F	2.8	20	g.6180502G>A c.1117C>T	p.Arg373*	Nonsense	Ex.10/Hom	NA	NA	0/6/251032 (All) 0/4/30550(SA)	rs62643625	Baronciani (2000) Thromb Haemost 84, 536
vWD38	11	F	2.1	15	g.6125368A>C c.5342T>G	p.Leu1781Trp	Missense	Ex. 31/Hom	PPh2 = PD SIFT = DL MT = P	*H. sapiens*	NR	NA	Ahmed, 2019
vWD39	17	M	1.8	14	g.6132925C>T c.3251G>A	p.Cys1084Tyr	Missense	Ex. 25/Hom	PPh2 = D SIFT = DL MT = DC	*Danio rerio* *(D. rerio)*	0/2/124535 (All) 0/2/30464 (SA)	rs759805079	Novel (This study)
vWD40	7	M	0.9	18	g.6184664delG c.712delG	p.His238Thrfs*219	Frameshift	Ex. 7/Het	NA	NA	NR	NA	Novel (This study)
vWD41	40	F	1.3	23	g.6085414G>A c.7300C>T	p.Arg2434*	Nonsense	Ex. 43/Hom	NA	NA	0/2/31380 (All) NR (SA)	rs62643640	Baronciani (2003) Blood Cells Mol Dis 30, 264 and others
vWD42	19	M	1.4	12	g.6220098A>T c.257T>A	p.Val86Glu	Missense	Ex. 4/Hom	PPh2 = D SIFT = DL MT = DC	*G. gallus*	NR	NA	Ahmed, 2019
vWD43	22	M	1.2	13	g.6184504delA c.871delT	p.Cys291Alafs*166	Frameshift	Ex. 7/Hom	NA	NA	NR	rs1565853817	Novel (This study)
vWD44	26	M	2.9	14	g.6094256G>A c.6931C>T	p.Arg2311Cys	Missense	Ex. 40/Hom	PPh2 = D SIFT = DL MT = DC	*Xenopus tropicalis* *(X. tropicalis)*	0/22/282886 (All) 0/1/30616 (SA)	rs150725355	Novel (This study)
vWD47	25	M	1.3	14	g.6132925C>T c.3251G>A	p.Cys1084Tyr	Missense	Ex. 25/Hom	PPh2 = D SIFT = DL MT = DC	*D. rerio*	0/2/124535 (All) 0/2/30464 (SA)	rs759805079	Novel (This study)
vWD48	26	M	2.7	11	g.6127545delA c.5039delT	p.Leu1680Profs*13	Frameshift	Ex. 28/Hom	NA	NA	NR	NA	Novel (This study)
vWD49	21	M	0.3	19	g.6085414G>A c.7300C>T	p.Arg2434*	Nonsense	Ex. 43/Hom	NA	NA	0/2/31380 (All) NR (SA)	rs62643640	Baronciani (2003) Blood Cells Mol Dis 30, 264 and others
vWD50	14	M	0.4	16	g.6180502G>A c.1117C>T	p.Arg373*	Nonsense	Ex.10/Hom	NA	NA	0/6/251032 (All) 0/4/30550(SA)	rs62643625	Baronciani (2000) Thromb Haemost 84, 536
vWD51	18	F	1.4	23	g.6132873G>T c.3303C>A	p.Cys1101*	Nonsense	Ex.25/Hom	NA	NA	NR	NA	Bowman (2013) J Thromb Haemost 11, 512
vWD53	21	F	1.1	21	g.6085414G>A c.7300C>T	p.Arg2434*	Nonsense	Ex. 43/Hom	NA	NA	0/2/31380 (All) NR (SA)	rs62643640	Baronciani (2003) Blood Cells Mol Dis 30, 264 and others
vWD54	23	F	2.2	17	g.6127609G>A c.4975C>T	p.Arg1659*	Nonsense	Ex.28/Het	NA	NA	0/16/281716 (All) 0/0/30562 (SA)	rs61750595	Zhang (1992) Hum Mol Genet 1, 61 and others
vWD55	27	M	1.5	17	g.6182812G>A c.970C>T	p.Arg324*	Nonsense	Ex.8/Hom	NA	NA	0/3/251370 (All) 0/2/30616 (SA)	rs61754000	Schneppenheim (1994) Hum Genet 94, 640 and others
vWD56	29	F	2.3	11	g.6092417_6092426del c.6977-6_6980del	p.Cys2327Thrfs*3	Frameshift & splice site	Ex. 41/Hom	Splice score—100%	NA	NR	NA	Novel (This study)
					g.6092415C>G c.6982G>C	p.Asp2328His	Missense	Ex. 41/Hom	PPh2 = D SIFT = DL MT = DC	*Ciona intestinalis* *(C. intestinalis)*	NR	NA	Novel (This study)
vWD57	15	F	1.5	9	g.6132925C>T c.3251G>A	p.Cys1084Tyr	Missense	Ex. 25/Hom	PPh2 = D SIFT = DL MT = DC	*D. rerio*	0/2/124535 (All) 0/2/30464 (SA)	rs759805079	Novel (This study)
vWD58	32	F	1.4	22	g.6180502G>A c.1117C>T	p.Arg373*	Nonsense	Ex.10/Hom	NA	NA	0/6/251032 (All) 0/4/30550(SA)	rs62643625	Baronciani (2000) Thromb Haemost 84, 536
vWD61	22	F	2.4	19	g.6166240T>G c.1730-2A>C	NA	Splice site	In. 14/Hom	Splice score—100%	NA	0/1/133334 (All) 0/1/21944 (SA)	rs61754014	Novel (This study)
vWD62	5	M	2.3	19	g.6094781G>T c.6849C>A	p.Cys2283*	Nonsense	Ex.39/Hom	NA	NA	NR	NA	Novel (This study)
vWD63	25	F	2.2	19	g.6125368A>C c.5342T>G	p.Leu1781Trp	Missense	Ex. 31/Hom	PPh2 = PD SIFT = DL MT = P	*H. sapiens*	NR	NA	Ahmed, 2019
vWD66	9	M	1.3	13	g.6128787G>A c.3797C>T	p.Pro1266Leu	Missense	Ex. 28/Hom	PPh2 = D SIFT = T MT = DC	*Mus musculus* *(M. musculus)*	1/234/281180 (All) 0/7/30468 (SA)	rs61749370	Holmberg (1993) J Clin Invest 91, 2169
vWD67	13	F	1.2	17	g.6181537A>G c.1069T>C	p.Tyr357His	Missense	Ex. 9/Het	PPh2 = D SIFT = DL MT = DC	*D. rerio*	NR	NA	Novel (This study)
					g.6184594_6184595insC c.780_781insG	p.Leu261Alafs*42	Frameshift	Ex. 7/Het	NA	NA	0/2/251064 (All) 0/0/30612 (SA)	NA	Novel (This study)
vWD68	12	F	1.3	15	g.6132948G>C c.3228C>G	p.Asp1076Glu	Missense	Ex. 25/Het	PPh2 = PD SIFT = T MT = DC	*M. musculus*	NR	NA	Novel (This study)
vWD70	9	F	1.7	12	g.6128787G>A c.3797C>T	p.Pro1266Leu	Missense	Ex. 28/Hom	PPh2 = D SIFT = T MT = DC	*M. musculus*	1/234/281180 (All) 0/7/30468 (SA)	rs61749370	Holmberg (1993) J Clin Invest 91, 2169
vWD71	20	F	1.8	17	g:6181574_6181575insG c.1031_1032insC	p.Glu344Aspfs*39	Frameshift	Ex. 9/Het	NA	NA	NR	NA	Novel (This study)

Table 1: All, gnomAD frequency data for all populations; D, probably damaging; DC, disease causing; DL, deleterious; Ex, exon; F, female; gnomAD v2.1.1, summary of data within Genome Aggregation database; H, homozygotes in gnomAD; h, heterozygous alleles in gnomAD; In, intron; M, male; NA, not available; NR, not reported; SIFT, Sorting intolerant from tolerant (http://sift.jcvi.org/, accessed on 23 February 2022); PPh2, Polyphen2-HumVar (http://genetics.bwh.harvard.edu/pph2/, accessed on 23 February 2022); MT, Mutation Taster (http://www.mutationtaster.org/, accessed on 23 February 2022); M, male; het, heterozygous; hom, homozygous; SA, gnomAD frequency data for South Asians; T, total alleles in gnomAD. The mutation coordinates were described according to the Human genome reference assembly GRCh37/hg19 (NM_000552.3, VWF). The gene symbol and sequence variants were defined according to the recommendations of Human Genome Organization (HUGO) human gene nomenclature committee, available at https://www.genenames.org/, accessed on 23 February 2022 and Human Genome Variation Society (HGVS) sequence variant nomenclature available at https://varnomen.hgvs.org/ and accessed on 23 February 2022, respectively.

## Data Availability

The data presented in this study are available in supplementary material.

## Supplementary Materials

The following supporting information can be downloaded at: https://www.mdpi.com/article/10.3390/genes13060971/s1, Figure S1: Workflow used in the study; Figure S2: *VWF* sequence annotated for target CpG sites (cg23551979 and cg04053108) that were analyzed for MSP based DNA methylation; Figure S3: Representative Sanger DNA sequencing confirmation electropherograms of missense mutations and nonsense mutations in vWD patients; Figure S4: Representative Sanger DNA sequencing confirmation electropherograms of frameshift mutations and splice site *VWF* mutations in vWD patients; Figure S5: Demographic, clinical and laboratory features of vWD patients across *VWF* mutation detection status; Table S1: Primer sequences for Sanger DNA sequencing confirmation of *VWF* mutations detected in this study; Table S2: Primers for synthesis (*VWF* MSP assay); Table S3A: *VWF* mutation detection frequency versus gender; Table S3B: *VWF* mutation detection frequency versus family history; Table S3C: *VWF* mutation detection frequency versus alloantibodies development; Table S3D: *VWF* mutation detection frequency versus bleeding time.
