# Supplementary material for: Genetic Alterations, DNA Methylation, Alloantibodies and Phenotypic Heterogeneity in Type III von Willebrand Disease
Source: Genes (Basel). 2022 May 28;13(6):971. doi: 10.3390/genes13060971 (PMC9222927; doi:10.3390/genes13060971)
Supplement: Supplementary file 1 [file genes-13-00971-s001.zip › genes-1718247-supplementary.pdf]

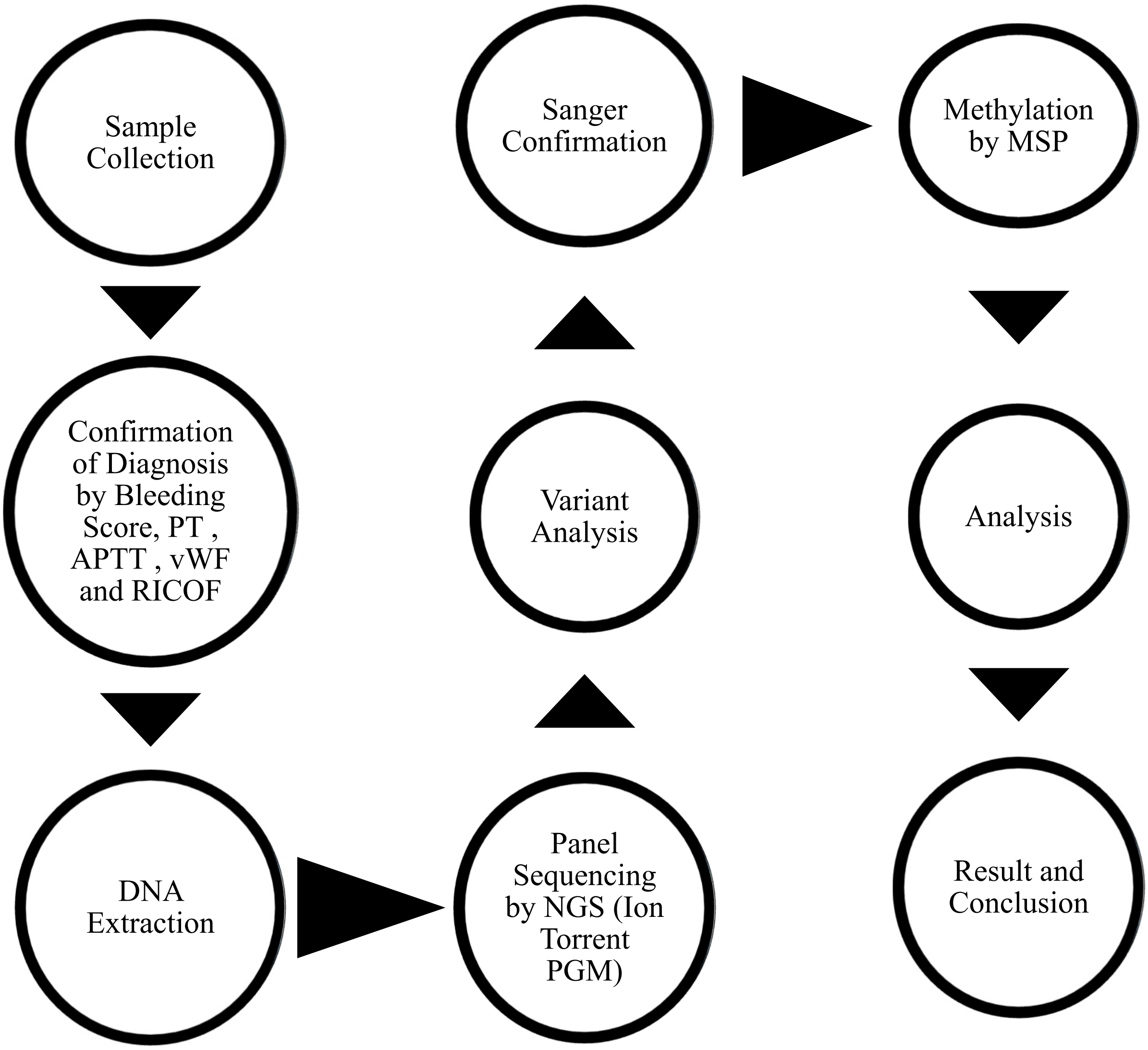

Supplementary Figure S1: Workflow used in the study

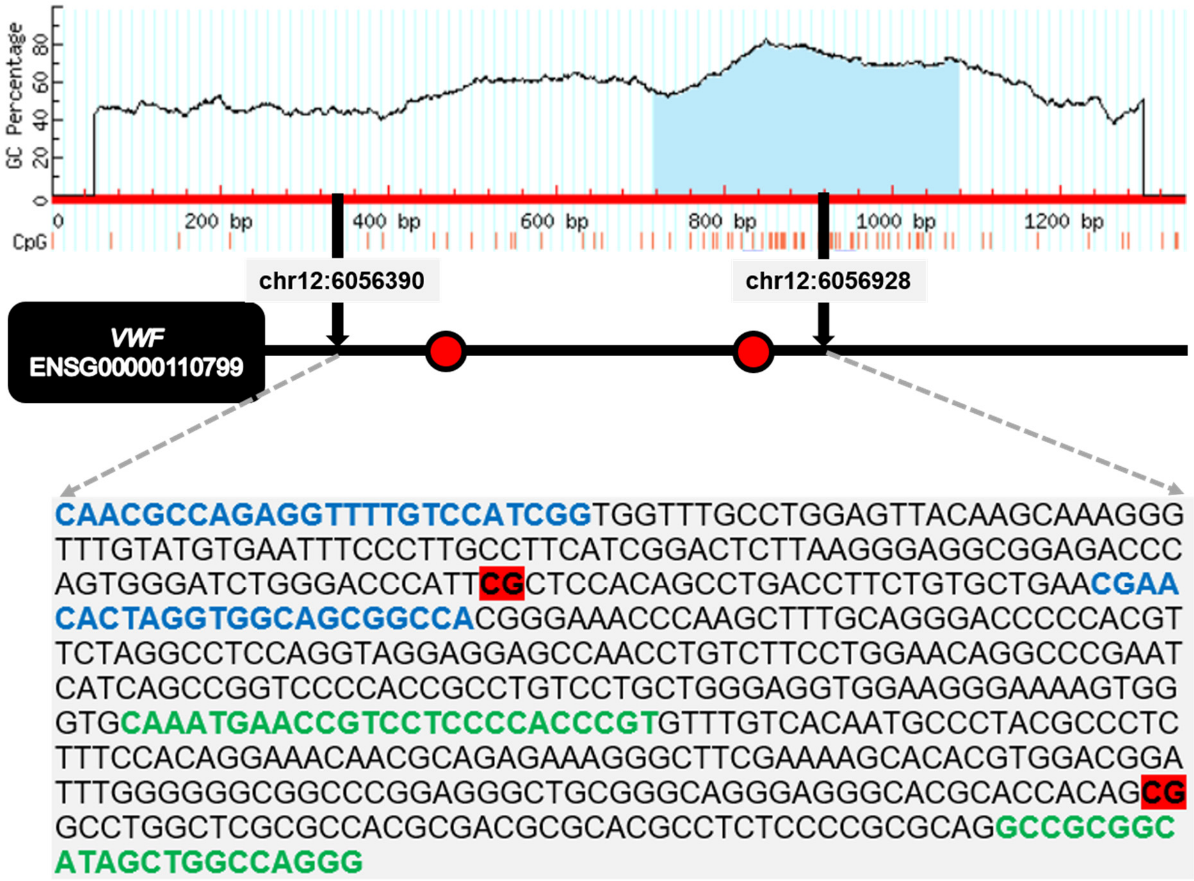

Supplementary Figure S2: *vWF* sequence annotated for target CpG sites (cg23551979 and cg04053108) that were analyzed for MSP based DNA methylation. The upper figure displays CpG sites density of this region with CpG island marked in blue (https://www.urogene.org/cgi-bin/methprimer). The location of target CpG sites is marked by red circles. In the genomic sequence below, the target CpG sites are highlighted in red, while MSP primer annealing sites for each of the CpG target sites are given in blue and green font.

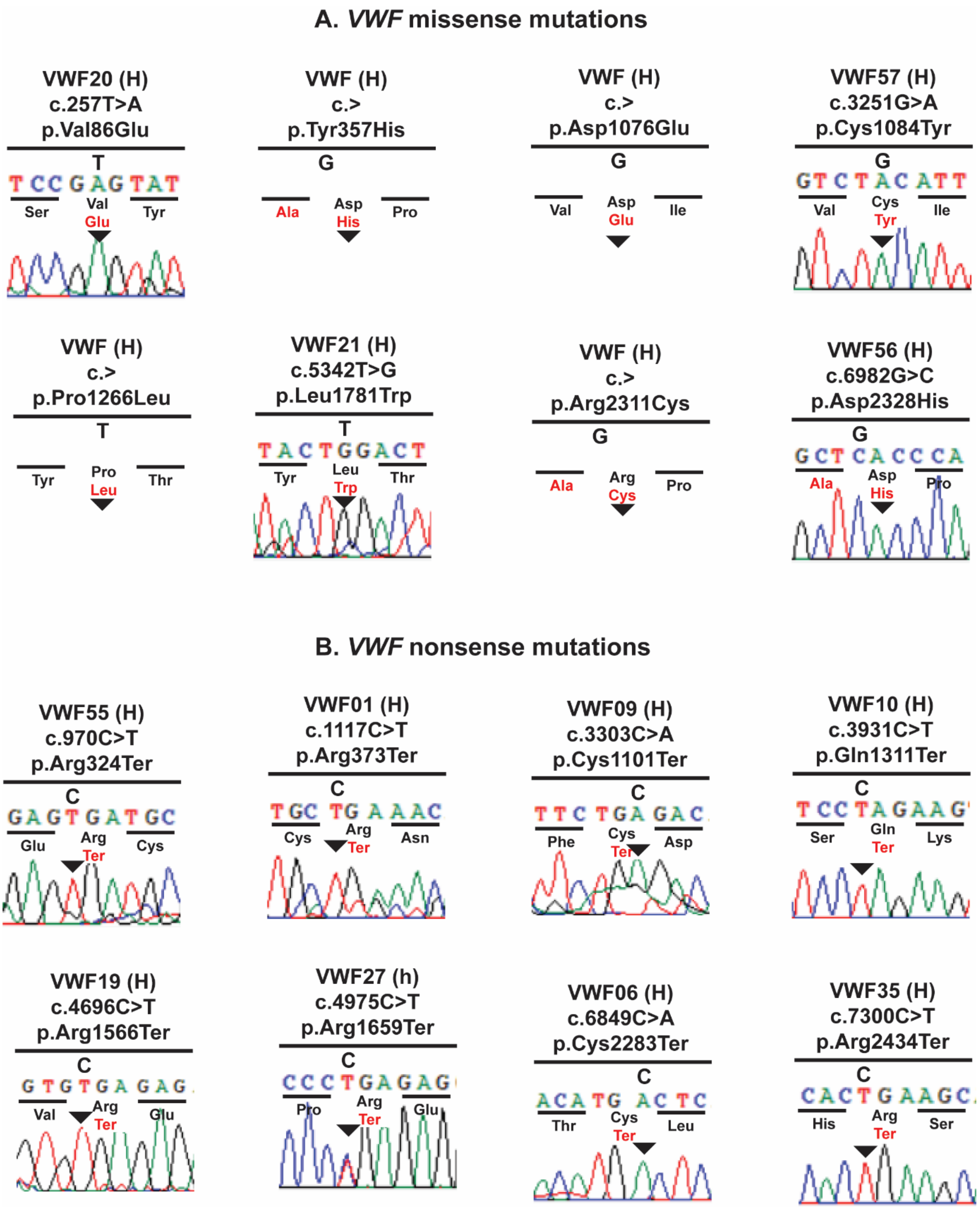

Supplementary Figure S3: Representative Sanger DNA sequencing confirmation electropherograms of **A.** missense mutations, and **B.** nonsense vWF mutations in vWD patients. Sample IDs are shown on top, and cDNA change (c.) and protein sequence change (p.) are shown below. Arrow heads indicate position of mutated nucleotide. (H), homozygous; (h), heterozygous.

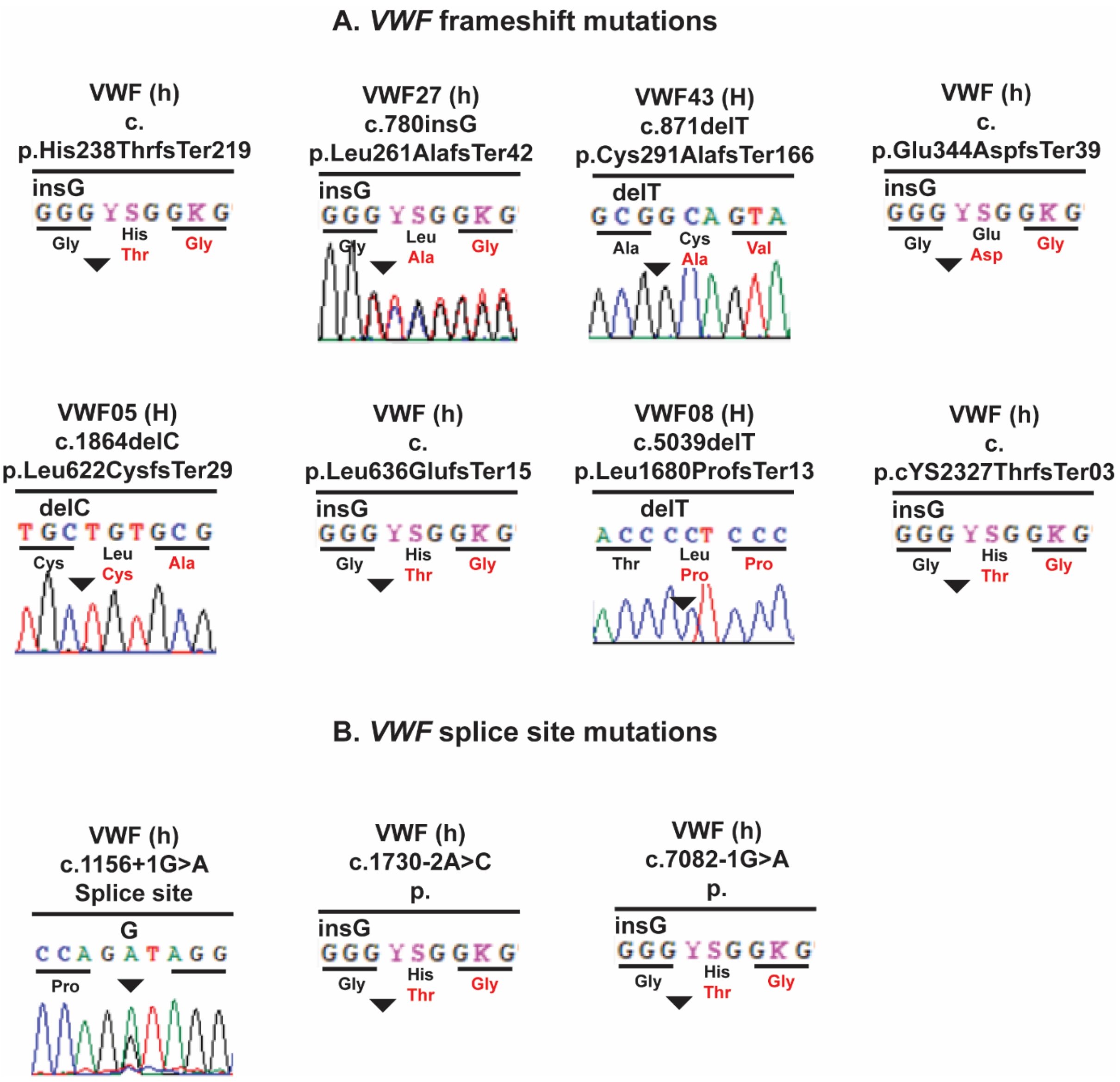

Supplementary Figure S4: Representative Sanger DNA sequencing confirmation electropherograms of **A.** frameshift mutations, and **B.** splice site vWF mutations in vWD patients. Sample IDs are shown on top, and cDNA change (c.) and protein sequence change (p.) are shown below. Arrow heads indicate position of mutated nucleotide. (H), homozygous; (h), heterozygous.

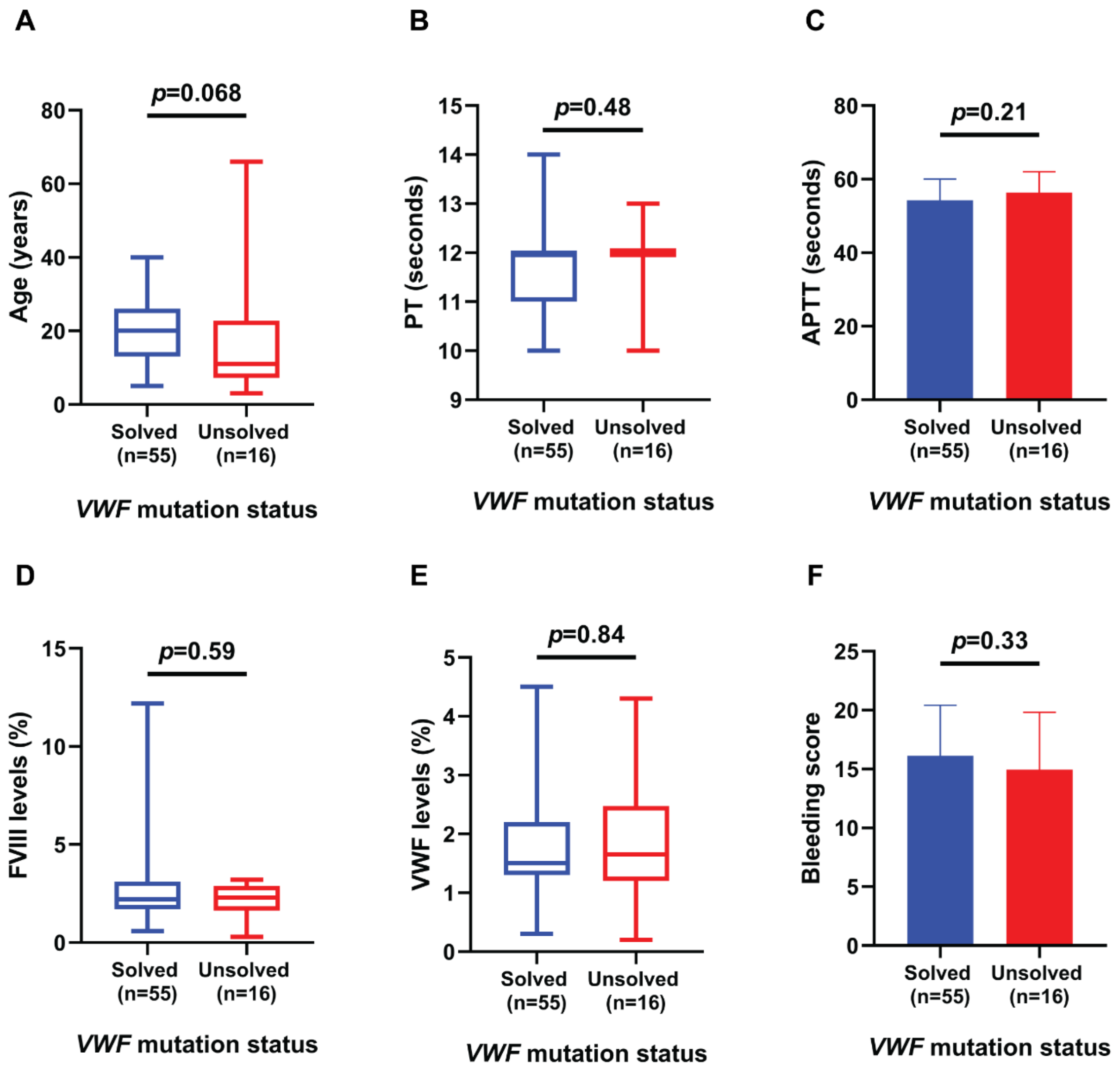

Supplementary Figure S5: Demographic, clinical and laboratory features of vWD patients across vWF mutation detection status including **A.** median age, **B.** PT, **C.** APTT, **D.** FVIII levels, **E.** vWF antigen levels, and **F.** bleeding score.

Supplementary Table S1: Primer sequences for Sanger DNA sequencing confirmation of *vWF* mutations detected in this study

Serial No.	Primer	Sequence (5'-3')	Size (bases)	Tm (Cº)	PCR product size (bp)	Representative mutations covered	Reference
1	vWF_Ex4_F	CCTTCTAACCCCAACCCCATGT	22	64.2	546	p.Val86Glu	Self-designed
2	vWF_Ex4_R	CCTGAGGGCAATGTATCCAGAA	22	62.1			
3	vWF_Ex7_F	GGGAGACACTAACGGAGCATA	21	61.2	399	p.His238Thrfs*219<br>p.Leu261Alafs*42<br>p.Cys291Alafs*166	Self-designed
4	vWF_Ex7_R	CTTTGTAGTCACTGGCTGGC	20	60.5			
5	vWF_Ex8_F	GACCTGGAAGCCTGAAGACA	20	60.5	400	p.Arg324*	Self-designed
6	vWF_Ex8_R	TCACGCTGGACAAAGACATTT	21	57.5			
7	vWF_Ex9_F	TGGAAGAAACCCAACCATTGTC	22	60.1	488	p.Glu344Aspfs*39<br>p.Tyr357His	Self-designed
8	vWF_Ex9_R	AGTTTGGAGGGACAGCTAGAGA	22	62.1			
9	vWF_Ex10_F	TTTCCCACATCCCTTCGTTTG	21	59.5	266	p.Arg373*	Self-designed
10	vWF_Ex10_R	CAACTTCTCGCTGCCTTGAG	20	60.5			
11	vWF_Ex15_F	CACTCCTCCCCACCACATC	19	61.6	370	p.Leu622Cysfs*29<br>p.Arg636Glufs*15	Self-designed
12	vWF_Ex15_R	CCCTACGCCCTCTTTCCAC	19	61.6			
13	vWF_Ex25_SN	CCAGACTAAGAGCCAGAGTTC	21	61.2	255	p.Asp1076Glu<br>p.Cys1084Tyr	18
14	vWF_Ex25_ASN	CATCTGAGAACATGAGGGC	19	57.5			
15	vWF_Ex28_1_SN	TCACTTGGATGTGGAATGGTCC	22	62.1	1036	p.Pro1266Leu<br>p.Gln1311*<br>p.Arg1566*<br>p.Arg1659*<br>p.Leu1680Profs*13	18
16	vWF_Ex28_1_ASN	AACTCCTTGCTCCTGTTGAAGTC	23	62.9			
17	vWF_Ex28_2_SN	ATGGTTCTGGATGTGGCGTTC	21	61.2	653		
18	vWF_Ex28_2_ASN	GTATCTTGGCAGATGCATGTAGC	23	62.9			
19	vWF_Ex31_SN	ACCGTTAAGACAGGGTGTCG	20	60.5	345	p.Leu1781Trp	18
20	vWF_Ex31_ASN	ACCAGGACAGAGGTTGGTAT	20	58.4			

21	vWF_Ex39+40_F	AGAATGTCCTGTGCCCTTTCT	21	59.5	786	p.Cys2283* p.Arg2311Cys	Self-designed
22	vWF_Ex39+40_R	TCCTTACCCACCTCCTTTCAC	21	61.2			
23	vWF_Ex41_F	ACCTTTCTGAACTCTGCATGGA	22	60.1	292	p.Cys2327Thrfs*3 p.Asp2328His	Self-designed
24	vWF_Ex41_R	TTCCAGATGTACTCCCAACCC	21	61.2			
25	vWF_Int41_F	AGCTGAATACTTACGAGCCCTG	22	62.1	458	c.7082-1G>A splice site mutation	Self-designed
26	vWF_Int41_R	TAGCACTTGGTTTGGGCAAGAA	22	60.1			
27	vWF_Ex43_F	GGGAAAGGAGGAGGACGAAAT	21	61.2	594	p.Arg2434*	Self-designed
28	vWF_Ex43_R	TCTTCTTACCCAGCCCTAACC	21	61.2			

Supplementary Table S2: Primers for synthesis (vWF MSP assay)

Sr. #	Primer	Sequence	Length	Tm	Product size
1	vWF_ cg23551979_M_F	TAACGTTAGAGGTTTTGTTTATCGG	25	60.9	179bp
2	vWF_ cg23551979_M_R	TAACCGCTACCACCTAATATTCG	23	60.9	
3	vWF_ cg23551979_U_F	TAATGTTAGAGGTTTTGTTTATTGG	25	57.6	179bp
4	vWF_ cg23551979_U_R	TAACCACTACCACCTAATATTCATT	25	59.2	
5	vWF_ cg04053108_M_F	GTATAGTTGGTTAGGGCGTCGT	22	62.1	159bp
6	vWF_ cg04053108_M_R	CAAATTCTCCGAAAAAACGTA	21	53.4	
7	vWF_ cg04053108_U_F	GGTATAGTTGGTTAGGGTGTTGT	23	60.9	159bp
8	vWF_ cg04053108_U_R	AACCAAATTCTCCAAAAAAACATA	24	55	

Supplementary Table S3A: vWF mutation detection frequency versus gender

**Gender**	**Detection of causative vWF mutation**		**OR (95% CI)**	***p*-value**
	Yes (n, %)	No (n, %)	0.58 (0.18-1.81)	0.51
Male	27 (49.1%)	10 (62.5%)		
Female	28 (50.9%)	06 (37.5%)		

Supplementary Table S3B: vWF mutation detection frequency versus family history

**Positive family history**	**Detection of causative vWF mutation**		**OR (95% CI)**	***p*-value**
	Yes (n, %)	No (n, %)	0.58 (0.16-2.05)	0.55
Yes	35 (63.6%)	12 (75%)		
No	20 (36.4%)	04 (25%)		

Supplementary Table S3C: vWF mutation detection frequency versus alloantibodies development

**Alloantibodies development**	**Detection of causative vWF mutation**		**OR (95% CI)**	***p*-value**
	Yes (n, %)	No (n, %)	NA	0.34
Yes	05 (10%)	00 (0%)		
No	50 (90%)	16 (100%)		

Supplementary Table S4D: vWF mutation detection frequency versus bleeding time

**Bleeding time**	**Detection of causative vWF mutation**	**OR (95% CI)**	***p*-value**

	Yes (n, %)	No (n, %)	NA	0.32
≥15min	49 (89.1%)	16 (100%)		
<15min	06 (10.9%)	00 (0%)		

Procedure of Hematological Tests:

P1: Platelet Count

Three ml venous blood in an EDTA vacutainer tube was analysed on Sysmex XT-1800i (Kobe, Japan) haematology analyzer within two hours of collection of the blood sample. Peripheral smears were made and stained with Giemsa stain of cases with low platelet count to rule out pseudo thrombocytopenia. The normal range was taken as 150 to 400 X 10^9/L.

P2: Bleeding Time (BT) By Modified Ivy's Technique

The patient was made to sit comfortably. A blood pressure cuff was put on the upper arm of the patient. With the help of the inflator, the cuff was inflated to 40 mm Hg. Afterwards, a standard incision of 1mm was made on the volar surface of the vein-free area on the forearm. The filter paper was used to draw off blood every 30 seconds until it stopped automatically. It depends upon the number and function of platelets, which adhere to the sub endothelium and form aggregates. The normal range is 3-7 minutes.

P3: Activated Partial Thromboplastin Time

P3.1 Principle

It determines how long test plasma takes to clot after contact factors have been activated without the use of tissue thromboplastin. This metric measures the overall efficacy of the intrinsic coagulation pathway. Factor XIIa is generated during incubation, and it cleaves Factor XI to XIa. After recalcification, Factor XIa activates Factor IX, and the coagulation process continues until a fibrin clot is produced.

P3.2 Method

APTT was determined manually using a water bath for incubation and manual readings. The normal reference range is 27 – 34 seconds.

P4 Factor VIII Level

P4.1 Principle

It is APTT based. The assay consists of measuring clotting time in the presence of APTT reagent (containing cephalin and activator), patient's platelet-poor plasma, and Factor VIII deficient plasma (all other clotting factors are present in excess except factor VIII).

P4.2 Method:

Factor VIII level was determined on Automated coagulation analyser Stago, using its reagent and Factor VIII deficient human plasma. The normal reference range is 50 to 150%.

P5: von Willibrand Factor Antigen Immunoturbidimetric assay.

P5.1 Principle

As the first step latex microparticles are incubated with plasma. These latex microparticles are coated with vWF specific antibodies for the immunoturbidimetric assay. If vWF antigen is present in the plasma, a reaction of vWF ag and vWF antibody will take place. This reaction leads to the agglutination of latex microparticles. In turn, agglutination of microparticles causes an increase in the turbidity of the solution. This agglutination results in increased turbidity of the solution. Turbidity, in turn, increases the absorbance. Photometric quantification is done for the absorbance of the solution. In the end, vWF: Ag concentration is calculated with the help of the standard curve.

P5.2 Method

von Willebrand factor antigen was determined on Stago analyser using Liatest reagent vWF:Ag. The normal reference range is 50 to 200%.

P6: Ristocetin Cofactor Assay

P6.1 Principle

The standard measure of vWF activity quantifies the ability of plasma vWF to agglutinate platelets via platelet membrane Glycoprotein Ib in the presence of Ristocetin. In the presence of Ristocetin, washed platelets do not agglutinate because they require vWF. When we add normal plasma platelets, they tend to agglutinate in the presence of ristocetin. Here normal plasma act as a source of vWF. The dose-response curve is obtained, which depends upon the amount of vWF added. Commercially available formalinized fixed, platelet preparations are used. This assay is one of the most sensitive and specific tests for functional assessment of vWF.

P6.2 Method

Ristocetin cofactor was determined on Aggregation Remote Analyser Module (AggRAM) Helena, Biosciences Europe using formalinized fixed platelets, Ristocetin, and Abnormal Ristocetin Cofactor Control. The normal reference range is 50% to 200%.

CONDENSED MCMDM-1 BLEEDING QUESTIONNAIRE:

Patient Information

Name ____

Address ____

____

Phone Number _______________ Email ___________________

Gender Male☐ Female ☐

Age ___________ Date of Birth ____________ (DD/MO/YYYY)

Ethnic Background _________________________

Presenting complaint of bleeding or bruising today Yes ☐ No ☐

Personal history of bleeding or bruising Yes ☐ No ☐

Ever been diagnosed with a bleeding disorder? Yes ☐ No ☐
Diagnosis: ____________________________

Family history of bleeding (at least one family member) Yes ☐ No ☐

If yes, what was the diagnosis? _____________________________
Pedigree:

Are you currently taking Oral Contraceptive Pills? Yes ☐ No ☐

If yes, brand name ________________________

Are you pregnant? __________ Gestation time __________

Specify any herbals and/or medications that you have taken in the past 30 days:

Name	Dose	Route	Frequency	Duration

Nosebleeds Yes ☐ No ☐

Number of episodes/year ☐ < 1 ☐ 6 - 12
☐ 1 – 5 ☐ > 12

Duration of average episode ☐ < 1 minute
☐ 1 – 10 minutes
☐ > 10 minutes

Medical attention ☐ Yes ☐ No

☐ Consultation only
☐ Cauterization/packing
☐ Antifibrinolytics
☐ DDAVP
☐ Transfusion/Replacement

Bruising Yes ☐ No ☐

Location ☐ Exposed sites
☐ Unexposed sites

Size of average ☐ < 1 cm
☐ 1 – 5 cm
☐ > 5 cm

Minimal or no trauma Yes ☐ No ☐

Medical attention Yes ☐ No ☐

If yes, please specify ______________________________

Bleeding from minor wounds Yes ☐ No☐

Number per year
- ☐ < 1
- ☐ 1 – 5
- ☐ 6 or more

Duration of average episode
- ☐ < 5 minutes
- ☐ > 5 minutes

Medical attention ☐ Yes ☐ No
- ☐ Consultation only
- ☐ Surgical hemostasis
- ☐ Blood transfusion/DDAVP/Replacement

Oral cavity bleeding Yes ☐ No ☐

- ☐ Tooth eruption
- ☐ Gums, spontaneous
- ☐ Gums, after brushing
- ☐ Bites to lip and tongue

Medical attention ☐ Yes ☐ No
- ☐ Consultation only
- ☐ Surgical hemostasis/Antifibrinolytic
- ☐ Blood transfusion/DDAVP/Replacement

Post-dental extraction Yes ☐ No ☐

- ☐ No bleeding in at least 2 extractions
- ☐ None done, or no bleeding in 1 extraction

Medical attention ☐ Yes ☐ No

- ☐ Consultation only
- ☐ Resuturing or packing
- ☐ Blood transfusion/DDAVP/Replacement

Gastrointestinal Bleeding ☐ Yes ☐ No

- ☐ Ulcer, portal hypertension, hemorrhoids
- ☐ Spontaneous
- ☐ Surgery/Blood transfusion/DDAVP/Antifibrinolytic

Surgery Yes ☐ No ☐

- ☐ No bleeding in at least 2 surgeries
- ☐ None done, or no bleeding in 1 surgery

Post-op medical attention ☐ Yes ☐ No

- ☐ Consultation only
- ☐ Surgical hemostasis/Antifibrinolytic
- ☐ Blood transfusion/DDAVP/Replacement

Menorrhagia Yes ☐ No☐

Duration of average menstruation _____ days

Duration of heavy menstruation _____ days

How often do you change your pads/tampons

on heaviest days ______ hours

on average days ______ hours

What type of feminine product do you use? (i.e. panty liner, super absorbency tampon etc.)

____

Medical attention ☐ Yes ☐ No

- ☐ Consultation only
- ☐ Pill use/Antifibrinolytics
- ☐ Dilatation & curettage
- ☐ Iron therapy
- ☐ Blood transfusion/DDAVP/Replacement
- ☐ Hysterectomy

Post-partum hemorrhage Yes ☐ No☐

- ☐ No bleeding in at least 2 deliveries
- ☐ No deliveries, or no bleeding in 1 delivery

Medical attention ☐ Yes ☐ No

- ☐ Consultation only
- ☐ D&C/Iron therapy/Antifibrinolytics
- ☐ Blood transfusion/DDAVP/Replacement
- ☐ Hysterectomy

Muscle hematomas Yes ☐ No☐

- ☐ Post-trauma, no therapy
- ☐ Spontaneous, no therapy
- ☐ Spontaneous or traumatic requiring DDAVP or Replacement
- ☐ Spontaneous or traumatic requiring surgical Intervention or transfusion

Hemarthrosis Yes ☐ No☐

- ☐ Post-trauma, no therapy
- ☐ Spontaneous, no therapy
- ☐ Spontaneous or traumatic requiring DDAVP or Replacement
- ☐ Spontaneous or traumatic requiring surgical Intervention or transfusion

Central Nervous System Bleeding Yes ☐ No☐

- ☐ Subdural, any intervention
- ☐ Intracerebral, any intervention

Other

____

Medical attention Yes ☐ No☐

- ☐ Consultation only
- ☐ Surgical hemostasis/Antifibrinolytic
- ☐ Blood transfusion/DDAVP/Replacement
